# Monocarboxylate Transporter 8 Deficiency: Delayed or Permanent Hypomyelination?

**DOI:** 10.3389/fendo.2020.00283

**Published:** 2020-05-13

**Authors:** Pieter Vancamp, Barbara A. Demeneix, Sylvie Remaud

**Affiliations:** UMR 7221 Molecular Physiology and Adaptation, Centre National de le Recherche Scientifique-Muséum National d'Histoire Naturelle, Paris, France

**Keywords:** Allan-Herndon-Dudley Syndrome, SLC16A2 gene, monocarboxylate transporter 8, oligodendrocyte, myelination and myelin repair, thyroid hormones, thyroid hormone analog, neurodevelopment

## Abstract

Monocarboxylate transporter 8 (MCT8) deficiency or the Allan-Herndon-Dudley Syndrome (AHDS) is an X-linked psychomotor disability syndrome with around 320 clinical cases described worldwide. *SLC16A2* gene mutations, encoding the thyroid hormone (TH) transporter MCT8, result in intellectual disability due to impaired TH uptake in the developing brain. MCT8 deficiency is a multi-organ affecting disease with a predominant neuronal cell-based pathology, with the glial component inadequately investigated. However, deficiency in myelin, a key component of white matter (WM) enabling fast nerve conduction, is a TH-dependent hallmark of the disease. Nevertheless, analysis of the myelin status in AHDS patients has led to conflicting interpretations. The majority of individual case studies reported delayed myelination, that was restored later in life. In contrast, *post-mortem* studies and high-resolution MRIs detected WM (micro-) abnormalities throughout adolescence, suggesting permanent hypomyelination. Thus, interpretations vary depending on methodology to investigate WM microstructure. Further, it is unknown whether the mutation within the MCT8 is linked to the severity of the myelin deficiency. Consequently, terminology is inconsistent among reports, and AHDS is occasionally misdiagnosed as another WM disorder. The evolutionary conserved TH signaling pathway that promotes the generation of myelinating oligodendrocytes enabled deciphering how the lack of MCT8 might affect myelinogenesis. Linking patient findings on myelination to those obtained from models of MCT8 deficiency revealed underlying pathophysiological mechanisms, but knowledge gaps remain, notably how myelination progresses both spatially and temporally in MCT8 deficiency. This limits predicting how myelin integrity might benefit therapeutically, and when to initiate. A recurrent observation in clinical trials is the absence of neurological improvement. Testing MCT8-independent thyromimetics in models, and evaluating treatments used in other demyelinating diseases, despite different etiologies, is crucial to propose new therapeutic strategies combatting this devastating disease.

## Introduction

The Allan-Herndon-Dudley Syndrome (AHDS, OMIM #300523) is a very rare, X-linked psychomotor disability syndrome first described in 1944, almost exclusively affecting boys (1). The clinical picture of AHDS is diverse, including muscle wasting, hypotonia, spastic paraplegia, and severe intellectual disability with an IQ generally lower than 30 (2). Patients often develop complications and are prone to infections, and consequently have a decreased life expectancy, although some have lived beyond the age of 60. A hallmark of the disease are abnormal thyroid hormone (TH) serum levels. AHDS patients have low thyroxine (3,5,3′,5′-tetraiodothyronine, or T_4_), high 3,5′,3′-triiodothyronine (T_3_) and borderline-increased thyroid stimulating hormone levels (3, 4).

Soon after the identification of monocarboxylate transporter 8 (MCT8) as a highly-specific transmembrane TH transporter (5), inactivating mutations in the human *SLC16A2* gene encoding MCT8 were linked to the altered TH levels detected in AHDS patients (3, 4), now often referred to as the MCT8 deficiency syndrome. Since then, several point mutations, translocations and deletions have been described in more than 320 diagnosed AHDS patients worldwide (6, 7), though many more are to be expected. It is estimated that up to 4% of the X-linked intellectual disability syndromes involve *SLC16A2* mutations (8).

MCT8 facilitates cellular influx and efflux of the mainly inactive (pro)hormone T_4_ and the bioactive T_3_. The uptake of lipophilic THs is a prerequisite for T_3_ to bind to nuclear TH receptors and act on gene transcriptional activity. The amount of available T_3_ to bind receptors is additionally controlled by deiodinases (DIOs), either activating T_4_ into T_3_ (DIO2), or inactivating T_4_ into reverse T_3_, or T_3_ into T_2_ (DIO3) (9). TH action is of vital importance for the development of many organs, including the central nervous system (CNS). Genome-wide studies in rodent brains revealed hundreds of TH target genes whose expression is directly and indirectly regulated (10, 11) through dynamic interactions of (un)liganded TH receptors with chromatin and DNA (12), as well as through epigenetic modifications (13). A strict spatiotemporal regulation of gene expression underlies the highly orchestrated development of the human CNS that occurs over a period of many years up to adolescence, and continues throughout life in some brain regions (14–17). Animal studies showed that THs orchestrate many cellular processes underlying the foundation of the CNS cyto-architecture (18, 19), including cell proliferation, cell fate decisions, migration and differentiation, but also maturation including axogenesis, synaptogenesis and myelinogenesis (20).

A key developmental process regulated by TH is myelination (21). Neuronal axons in the vertebrate CNS with a diameter > 0.4 μm are encapsulated by myelin enabling faster electrical conduction over relatively long distances (22). Any damage to the white matter (WM) has devastating consequences for neural connectivity, impairing neurological performance and motor-coordination (23). Myelin is a lipid-rich (fatty) substance, giving it a white appearance in *post-mortem* tissue, hence the name WM for myelinated CNS regions. In humans, the peak of myelination occurs primarily during the first 2 years after birth, and is quasi complete at 5 years of age, but continues until early adulthood in some regions like the frontal cortex (24). Myelination follows a fixed temporal-spatial pattern, with axons in the brain stem being the first to be myelinated, followed by those in the cerebellum, and finally the cerebral cortex (25, 26). Compact myelin is a hallmark of the CNS of craniates that allowed the expansion and diversification of the vertebrate subphylum (27, 28). Furthermore, it is a well-known TH-dependent process, and associates with a neonatal peak in TH levels detected in all vertebrates (29). Accordingly, congenital hypothyroidism, resulting from an ineffective thyroid gland at birth, is characterized by delayed myelination. Treating new-borns with levothyroxine can restore myelination progress (30), showing that TH is a key player modulating myelin integrity.

Impaired myelination is also a common trait in AHDS patients, the majority of them having an abnormal WM content during the 1st years after birth (31, 32). However, as discussed herein, conflicting interpretations among case reports and brain imaging analyzes, reporting the myelin deficiency as either delayed or permanent, impede the unequivocal classification of AHDS within the broad family of demyelinating disorders. Consequently, there is sometimes an inconsistent use of terminology in different studies. A detailed description of the myelin phenotype is still missing in patient's later life, but also in animal models that replicate various aspects of the human disease. This complicates correct diagnosis of new AHDS patients and hampers prognosis of the extent to which the deficit in myelin might benefit from a potential treatment. Several MCT8-independent thyromimetics, currently tested to combat the peripheral thyrotoxicosis due to the excess of circulating T_3_ levels, might also improve myelination if their cellular mode of action is thoroughly understood, and if they can be administered early enough to obtain a maximal response.

Here, we review recent literature data to clarify the myelin deficit associated with AHDS and models of MCT8 deficiency. We also discuss potential mechanisms to investigate how the lack of MCT8 could hamper myelination, and we provide insights on MCT8-independent TH analogs and other drugs as promising candidates for myelin repair therapies.

## Clinical Data From Allan-Herndon-Dudley Syndrome Patients: Permanent or Delayed Myelination?

To classify AHDS correctly within the broad group of demyelinating disorders, it is imperative to use correct terminology. Clinical myelin disorders have been subdivided according to WM appearance and developmental progression, mostly assessed using brain magnetic resonance imaging (MRI) (26, 31). A leukoencephalopathy is any clinical neurological disorder characterized by abnormal development or degeneration of WM in the CNS. In particular, a leukodystrophy is a congenital leukoencephalopathy, i.e., with a genetic etiology (25). The fact that myelination primarily takes place during the first 2 years of postnatal life means it is important to determine whether a congenital myelination deficit is permanent or delayed. If the same pattern of myelination is observed in at least two MRIs, taken > 6 months apart in a child older than 1 year, the paucity is considered permanent and referred to as “permanent hypomyelination” (26). Whenever progress in myelination or WM content can be observed within this time interval, it is defined as “delayed myelination” (26).

During the Delphi convention in 2015, leukodystrophy experts redefined and subdivided the WM disorders into two new groups (31, 33). The first group comprises the “leukodystrophies.” These are defined as WM disorders, with glial cells being the main cause of the pathology and myelin sheath abnormalities the most apparent feature. The second group are the “genetic leukoencephalopathies” (gLEs), characterized by neuronal cell pathologies and/or by other systemic disease manifestations, whereby WM abnormalities are subordinate to the neurological component of the disease (31, 33).

**Figure 1:**
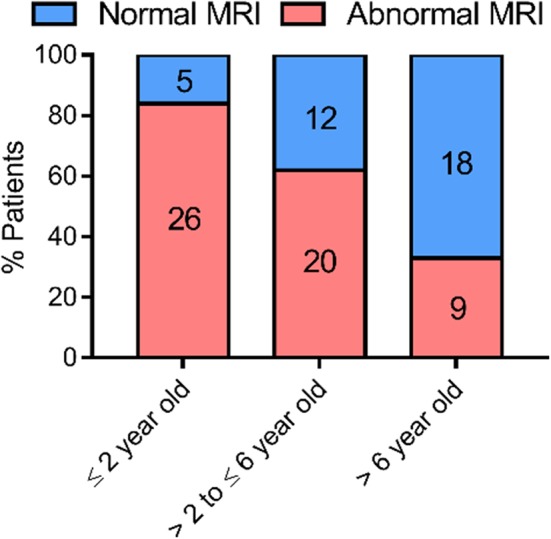
Evolution of myelination based on MRI data obtained from case-reports of 90 AHDS patients. The WM status as observed in the last MRI of each patient was used for the analysis. Sequential MRIs were not taken up in the analysis, so that each data point represents one patient. We ascribed a patient to the group “normal MRI” whenever the report specifically mentioned a normal WM status or the complete absence or disappearance of WM abnormalities in the last exam. In case a delay of any degree was reported or WM status showed abnormalities, we ascribed the patient to the group “abnormal MRI.” The references for the patient reports used for this analysis can be found in Table 1. This graph was made using GraphPad Prism v7.00.

To determine whether myelination is delayed or permanent in AHDS patients, data on WM status and progression is needed. Azzolini et al. performed a meta-analysis of the myelin status in 32 diagnosed AHDS patients from January 2004 until December 2012, and also reported a new case (34). We build on this list, citing cases found up until December 2019 and taking case reports into account that described the WM phenotype. We employed a similar search strategy to (34) using the following search terms: “MCT8” and “mutation,” “SLC16A2” and “mutation,” “Allan–Herndon–Dudley syndrome,” “AHDS” and “case report,” “AHDS” and “MRI” and “myelination.” We found an additional 18 peer-reviewed articles reporting on 61 patients for whom MRI was used to assess myelin status in the brain (Table 1). Hence, we had MRI data on 94 AHDS patients in total, representing roughly a quarter of all known cases. We only included data on a patient's last MRI to simplify the analysis (Figure 1). Four patients from Remerand et al. (32) were excluded, since it was unclear whether the WM profile was normal or abnormal for their last MRI. We divided the remaining 90 patients in groups younger than 2 years old (2 year-olds included), between 2 and 6 years old (6 year-olds included), and those older than 6 years. Two years was chosen as a first cut-off point based on (26), stating that it is difficult to evaluate a myelination deficit before the age of 2 years when the myelination process is still ongoing. The age of 6 years was chosen as an arbitrary cut-off given that myelination is virtually complete by 5 years (24).

**Table 1:** MRI data on case-reports of AHDS patients from October 2004 to December 2019.

**References**	**Patients**	**Mutation**	**First MRI (age)**	**Sequential MRI(s) (age)**
Dumitrescu et al. (3)	2	c.1212delT c.1535T>C	Normal myelination (2 y) Normal myelination (3 y)	N.d. N.d.
Holden et al. (35)	1	p.insI189	Abnormal myelination (3 m)	Delayed myelination (12 m, 22 m)
Kakinuma et al. (36)	1	c.485T>C	Abnormalities in the left putamen (3 y)	Progressive atrophy of WM (6 y)
Schwartz et al. (37)	1	c.703G>A	Normal myelination (13 y)	N.d.
Namba et al. (38)	1	c.1649delA	Extensive myelination delay (11 m)	Myelination proceeds slowly (4 y 2 m)
Papadimitriou et al. (39)	1	p.P537L	Delayed myelination of subcortical WM and thalamus (11 m)	N.d.
Sijens et al. (40)	1	c.1690G>A	WM hypoplasia, small CC (8 m)	WM hypoplasia, but local improvements (28 m)
	1	p.delF501	WM hypoplasia, small CC (10 m)	Myelination near normal (17 m)
Fuchs et al. (41)	1	c.812G>A	Infarction in left putamen, but normal WM (6 m)	N.d.
Vaurs-Barrière et al. (42)	1 1 1 1 1	Del exons 2-3-4 “ c.661G>A “ c.1558C>T	Normal myelination (10 y) Diffuse hypomyelination (4 y) Diffuse hypomyelination (6 y) Diffuse hypomyelination (1 y) Diffuse hypomyelination (6 m)	N.d. N.d N.d N.d Progressive improvement, but hypomyelinated periventricular tracts (5 y)
	1	c.1003C>T	Diffuse hypomyelination (14 m)	Normal myelination (10 y)
	1	c.962C>T	Abnormal myelination (13 m)	N.d.
	1	c.1826delC	Diffuse hypomyelination (5 y)	Diffuse hypomyelination (7 y)
Boccone et al. (43)	1	c.1343_1344insGCCC	Delayed myelination of semioval centers, normal spinal cord (5 y)	N.d.
Gika et al. (44)	1	Del exons 2-6	Marked myelination delay (21 m)	Myelination gradually improved but some delay was still present (30 m, 4 y)
	1	c.1306delT	Significant myelination delay (2 y, 9 m)	Little progression of myelination (3 y 7 m)
	1	c.683-5delTCT	Significant myelination delay (3 y, 9 m)	N.d.
	2	c.962C>T	Significant myelination delay in the genu and anterior limb of internalcapsule (in both cases at 9 m)	N.d.
Crushell and Reardon (45)	1	c.1614dupC	Delayed myelination, small CC (23 m)	N.d.
Tsurusaki et al. (46)	1 1	c.1102A>T	Delayed myelination (2 y) Normal myelination (8 y)	Normal myelination (13 y) N.d.
Zung et al. (47)	1	Del exons 2-6	Delayed WM myelination, small CC (6 m)	N.d.
Tonduti et al. (48)	1 1 1	c.1412T>C “ c.656G>A	Delayed myelination (20 m) Delayed myelination (11 m) Considered normal (4 m)	Almost normal myelination (5 y 6 m) N.d. Slow progression but still delayed WM myelination (2, 3, 4 y)
Boccone et al. (49)	2	c.670G>A	Generalized delayed myelination (3 y) Normal myelination (36 y)	N.d. N.d.
Azzolini et al. (34)	1	c.1251_1252insG	Normal Myelination (14 m)	N.d.
Anik et al. (50)	2	Del exons 3-4	Relatively normal myelination (6 m) Delayed myelination (6 y)	N.d. N.d.
Kobayashi et al. (51)	1 1	c.1621G>T “	Delayed myelination (26 y) Delayed myelination (13 y)	N.d. Some progression, but still immature myelination, cerebral WM atrophy (18 y)
	1	“	Delayed myelination (3 y)	N.d.
	1	“	Delayed myelination (11 y)	N.d.
López-Espíndola et al. (52)	1 1	p.L494P p.Q96X	Low or absent brain MBP signal (GW 30)^*^ Pale MBP staining, delayed myelination (11 y)^*^	N.d.^*^ N.d.^*^
Rodrigues et al. (53)	1	c.735_760dup	Delayed myelination (2 y)	N.d.
Yamamoto et al. (54)	1 1	c.1390_1392delCCC c.97T>C & c.449C>T	Marked myelination delay (7 m) No marked abnormalities (8 m)	N.d. Delayed myelination (17 m)
Armour et al. (55)	1 1	c.869C>T	Delayed myelination (23 m) Delayed myelination, small CC (9 m)	Normal myelination (10 y 10 m) N.d.
Gagliardi et al. (56)	1	c.652+1G>A	Delayed myelination (5 y)	N.d.
Kim et al. (57)	1	c.671C>T	Hypomyelination & decreased periventricular WM volume (9 m)	N.d.
La Piana et al. (58)	1	p.L291R	Significant myelination delay (7 m)	Mild progression (14, 26 m)
Matheus et al. (59)	5	N.d.	Delayed myelination, diffuse regions with more pronounced hypomyelination (8 m)	Progression to normal myelination in CC and corticospinal tracts in 3/5 cases (1, 2, 5, 5, 7 y^&^), but micro-abnormalities in other WM tracts remained in all cases N.d.
	1	N.d.	Delayed myelination (2 y)	
Bedoyan et al. (60)	2	c.321_322delTG	Delayed myelination, smaller CC (9 m)Delayed myelination, smaller CC (1 y)	N.d.N.d.
Charzewska et al. (31)	1	c.940C>T	Diffuse hypomyelination (20 m)	Strong improvement, except for periventricular WM tracts (6 y 1 m)
Ono et al. (61)	111	c.1333C>Ac.587G>Ac.1063_1064insCTACC	Delayed myelination (1 y)Delayed myelination (1 y 7 m)Delayed myelination (20 m, 3 y)	Normal myelination (8 y)Normal myelination (20 y)Normal myelination (21 y)
Shimojima et al. (62)	11	c.365G>Tc.661G>A	Delayed myelination (2 y 6 m)Marked hypomyelination (6 m)	Normal myelination (6 y)N.d.
Novara et al. (63)	111	c.812G>Ac.1690G>Ac.1691G>A	Delayed myelination (2 y)Moderate myelination delay, small CC (2 y) Normal myelination (8 y)	N.d.N.d.N.d.
Rego et al. (64)	1	c.1384G>A	Normal myelination (11 m)	Marked cerebral and cerebellar myelination delay (3 y)
Masnada et al. (65)	11	c.715A>Cc.1625T>C	Normal myelination (29 y)Delayed myelination (12 m)	N.d.Normal myelination (4 y 6 m, 7 y)
Remerand et al. (32)^+^	12	c.1621+2T>C c.1333C>T c.277C>T Del exons 2-3-5-6c.608T>C c.1412T>G c.1393-1G>C c.373InsCT c.1690G>A c.812G>A c.1406T>C c.1412T>C	Hypomyelination^#^	Myelination improvement in 12/19 cases, periventricular WM abnormalities in 4/12 (2 y 1 m – 14 y 5 m)
	5	Del exon 1 c.575A>G c.811C>A c.1621+2T>C c.1321T>C	Hypomyelination^#^	No improvement in 5/17 cases (6 m – 12 y 1 m)
	12	c.1412T>C c.1202G>A c.1691G>A	Hypomyelination (11 m)Normal myelination^#^	N.d.Normal myelination (8 y) Normal myelination (13 y 6 m)

We only provide patients in which the myelin status has been described, his/her age at MRI, and the corresponding gene mutation. When the latter was not available, we gave the protein mutation. CC, corpus callosum; GW, gestational week; m, months; MBP, myelin basic protein; N.d., not determined; WM, white matter; y, years.

* Post mortem study;

# No data on the age of examination;

& Age at last MRI;

+ *Unclear for 4/24 cases whether improvement was seen or not, and therefore not taken up in the table*.

Abnormal or delayed myelination was evident in 84% of the patients ≤ 2 years of age. Of the AHDS patients with MRI data that were older than 2 and ≤ 6 years of age 63% displayed some form of myelination delay. The percentage was reduced to 33% in patients older than 6 years (Figure 1), although the latter group consisted of only 27 patients. Thus, there tends to be a very slow but gradual improvement in myelin status in the patient cohort during this developmental period. A gradual progression in WM status was also observed in several of the patients that underwent sequential MRIs (Table 1). This finding defines MCT8 deficiency as a special case. Pouwels et al., with their long-time experience of demyelination studies, suggested that whenever deficient myelination is still observable in 2 year olds, it seems unlikely a child will ever display a normal WM content (26). The observed progression is developmental in nature and not a case of spontaneous regeneration of myelin, since no partially remyelinating sites, also called shadow plaques, were observed that are otherwise typical in myelinating disorders such as Multiple Sclerosis (MS) (66).

**Figure 2:**
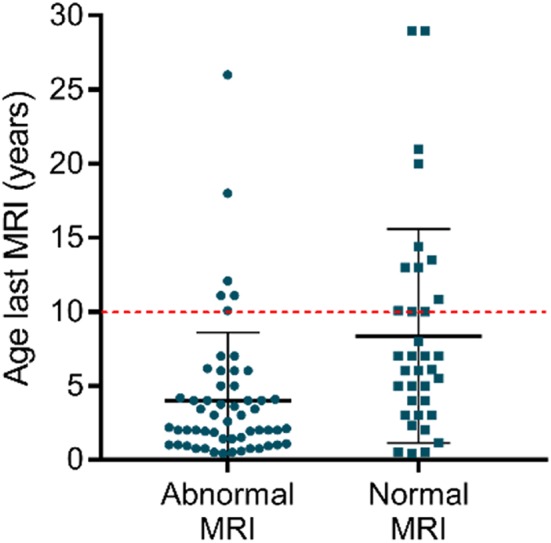
Myelin appearance (abnormal MRI vs. normal MRI) in AHDS patients in function of the age at the last MRI. Each point represents one patient, and the scatter plots also depict mean ± standard deviation. Finding normal WM status on MRI is more likely in older patients. Notice that above the age of 10 (red dotted line), there are still several patients with an abnormal WM status as observed on MRI. The references for the patient reports used for this graph can be found in Table 1. This graph was made using GraphPad Prism v7.00.

Remarkably, some patients never acquire a completely normal WM content, even after several years. Even though older patients more likely have normal myelination, some MRI data show no normalization in patients above the age of 10 [e.g., (32, 51)] (Figure 2). Clear signs of hypomyelination were also found during a *post-mortem* histological exam of the brain of a deceased 11-years-old AHDS boy (52). Inadequate myelination also coincides with several functional outcomes observed in AHDS patients, such as muscle dystonia, spastic paraplegia with poor head and limb control (32). A previous comparison of MRI data even identified three patients with a worsening myelin phenotype (34), and Remerand et al. also described three deteriorating cases out of their 24 patient cohort (32). The two patients examined in Anik et al. clearly illustrate the discrepancy among patients as a function of age (50): the two boys of the second investigated family are 6 months and 6 years old, but only the older boy displays hypomyelination.

In this last case, the myelin status differs extensively despite the brothers sharing the same 2.8 kb deletion in the *SLC16A2* gene that results in a complete loss of MCT8 uptake activity due to the lack of exons 3 and 4 (50). This case prompts the question whether or not a genotype-phenotype relationship exists between the type of gene mutation, its impact on MCT8 protein structure and function, and the severity of the myelin deficiency. *In vitro* cultures using human-derived fibroblasts, or JEG3- and COS9-cells previously identified several point mutations that resulted in residual TH uptake activity. These mutations are often associated with milder neurocognitive phenotypes in patients [e.g., (37, 65, 67)]. Other point mutations affecting proper substrate binding, trafficking or folding [e.g., (68, 69)], as well as large deletions, substitutions or premature stop-codons, render the MCT8 protein completely inactive, and result in severe mental and motor disabilities [e.g., (50, 70)].

Remerand and co-workers determined that for a group of AHDS patients for which radio- and neurological features were available in literature, 27% of them presented with moderate-to-mild intellectual disability, irrespective of age (32). However, how the type of mutation in each of these patients differentially affects MCT8 uptake activity is unknown. A genotype-phenotype relationship is expected for MCT8 mutations and the neurocognitive outcome (71), but a general trend has yet to be uncovered (6). Data on WM status in particular are currently too fragmentary to identify a potential link with the kind of mutation and the severity of the myelin deficiency.

More detailed MRI examinations also revealed that while the general WM structure seems intact as a whole, subtle localized myelin abnormalities might go unnoticed. Matheus and colleagues found a normally myelinated corpus callosum in three 6-years-old AHDS patients, but distinct tracts remained inadequately myelinated (59). The hypomyelination of subcortical U-fibers and periventricular tracts even became more discernible (59). This is corroborated by several other reports mentioning local, more subtle WM abnormalities too, as for instance in the periventricular WM tracts (31, 32, 42) and indicates regionally hypomyelinated areas may still be present in later life. Similar microstructural WM abnormalities were also observed in children with severe congenital hypothyroidism (72). This indicates that a global brain MRI is insufficient to discern between “normal” vs. “abnormal” myelination, and advocates a high-resolution and regional examination of the myelin status of the entire brain, and by extension the spinal cord.

Future studies employing a more accurate exploration of the WM phenotype by MRI imaging at regular time intervals would reveal if, and how fast exactly myelination improves in AHDS patients. Whenever possible, additional *post-mortem* analyses should include accurate WM examinations. Clinical data from newly discovered patients extend the database, and could unmask subgroups responding differently to MCT8 deficiency, or could uncover mutations harboring individual-specific traits related to myelination, or even other clinical features. An important observation is that in the patients that do exhibit a gradual increase in myelination, neurological functions do not improve. This suggests persistent neuronal problems throughout the CNS as being the main cause of abnormal sensorimotor functioning (58), although one can also not rule out a significant impact of poor feeding and extensive muscle wasting (37). Here too, additional MRI imaging examining the spinal cord WM should be performed to rule out any problems in electrical conductance along downstream axonal tracts. Moreover, novel brain imaging techniques such as positron emission tomography (PET) used to image progression of WM lesions in MS (73) could provide new ways to dynamically follow up myelination.

The enigmatic myelin phenotype accompanying MCT8 deficiency impedes the unequivocal classification of AHDS into a distinct group of WM disorders (Table 2), and it leads to unintentional inconsistencies in literature. For instance, Charzewska et al. proposed to classify it as a leukodystrophy (31), whereas Barkovich et al. did not include it in their list of “hypomyelinating disorders” (74), while Remerand et al. described the myelin deficit as hypomyelination (32). Furthermore, the unawareness of changing clinical terminology has led to occasionally mixed use of the terms “delayed” and “hypomyelination” in animal research too (see section myelin deficiency in animal models of MCT8 deficiency: permanent or delayed?). At the Delphi convention, AHDS was categorized as a gLE (31, 33). This classification is justified if delayed myelination is indeed a typical hallmark during the 1st years of development, but eventually becomes a non-pathognomonic trait if myelin formation recovers.

**Table 2:** Characteristics of AHDS to classify it as a genetic leukoencephalopathy (gLE) vs. a leukodystrophy.

**Traits in favor of a gLE**	**Traits in favor of a leukodystrophy**
Slow but gradual progression in brain myelination	Persistent hypomyelinated features in some patients
WM abnormalities subordinate to neuronal problems	WM micro-abnormalities found at later age
Neuronal cells are main cause of pathology	Endothelial and glial cells also affected
Non-CNS organs affected	No obvious neurological damage on MRI

*CNS, central nervous system; MRI, magnetic resonance imaging; WM, white matter*.

That would also mean that AHDS differs from the group of leukodystrophies displaying permanent hypomyelination without any progress noticeable on sequential MRIs (31, 75). An example is Pelizaeus-Merzbacher Disease (PMD) most often caused by duplications in the *PLP1* gene that encodes two primary components of myelin. In PMD, brain regions are permanently hypomyelinated and patients suffer from muscle hypotonia, ataxia, and speech problems (76).

However, 11% of patients originally diagnosed with PMD had mutations in the *SLC16A2* gene (42), indicating WM status itself is insufficient as a diagnostic marker. In addition, while AHDS is considered primarily as a neuronal cell-based pathology, a criterion for classifying it as a gLE, the glial component remains poorly investigated. It is however almost certain that glial cell types are affected in MCT8 deficiency (see section discovering underlying mechanisms: the oligodendroglial cell and the lack of MCT8), which is a typical hallmark of a leukodystrophy (Table 2). It might therefore be diagnostically more interesting to characterize AHDS as a hypomyelinating disorder (31), allowing it's integration into a new proposed classification system for leukodystrophies (77). As such, severe cases of myelin deficiency or ambiguous MRIs will require additional genetic and serum TH level testing as conclusive diagnostic criteria to discriminate between AHDS and other WM disorders such as PMD (78). That accelerates finding a diagnosis for many undiagnosed patients (78), but is also crucial to anticipate on how and when to therapeutically intervene. An earlier intervention might increase chances of a maximal response (26, 79).

## Discovering Underlying Mechanisms: The Oligodendroglial Cell and the Lack of MCT8

A crucial aspect of vertebrate brain evolution was the appearance of myelin, enabling fast electrical conduction across axons spanning increased distances with CNS expansion (28). TH levels peak during neonatal stages (29) during which myelination processes occurs (21, 27). The evolutionary conserved TH signaling pathways are thus an asset for studying how MCT8 deficiency might hamper TH-dependent myelination in animal models and AHDS patients.

### Thyroid Hormone Action in Oligodendroglial Cells

In the CNS, myelin is produced by oligodendrocytes, a glial cell type that also provides trophic support for neurons (80). While myelination occurs mainly postnatally in vertebrates, the pool of oligodendrocyte precursor cells (OPCs) is established during pre- and perinatal development. The premature CNS consists of multipotent neural stem cells that generate a pool of OPCs (gliogenic wave) after the neurogenic wave (29). These OPCs can further differentiate into oligodendrocytes, which can mature into fully functional, myelinating oligodendrocytes.

Decades of research revealed that TH action on OPCs and oligodendrocytes is strongly conserved [for a comprehensive review, see (80)]. Classic experiments in the 90s using *in vitro* OPC cultures derived from optic nerves collected from neonatal rats showed that T_3_ inhibits OPC proliferation in the presence of mitogens, induces differentiation into mature oligodendrocytes, and facilitates the formation of myelin extensions (81–85). Subsequent experiments showed that T_3_ stimulates OPC cell cycle exit by binding to the increasingly expressed TH receptor α1, while its interaction with TH receptor β induces timely expression of genes crucial for normal oligodendrogenesis and myelinogenesis (86–88). Important direct target genes include myelin basic protein (*MBP*), proteolipid protein (*PLP*) and Krüppel-like factor 9 (*KLF9*) (89, 90).

### Temporal Aspects of Thyroid Dependent Myelination *in vivo*

The crucial role of TH in promoting oligodendrocyte differentiation *in vitro* echoes the *in vivo* need of adequate TH levels for proper oligodendrogenesis in the CNS. Induction of hypothyroidism in postnatal rodents reduced oligodendrocyte numbers and delayed oligodendrocyte maturation, leading to insufficient myelination in several brain regions such as the anterior commissure and the corpus callosum (82, 91, 92). A recent study showed that both transient postnatal (from postnatal day 0 – 21) and permanent hypothyroidism in methimazole-treated rats decreased the number of myelinated axons in the anterior commissure at postnatal day 150, while the number of unmyelinated axons slightly increased (93). Since prenatal hypothyroidism in rats did not alter the total number of axons in the anterior commissure (94), it can be concluded that the ratio of unmyelinated vs. myelinated axons increased. The axons that were myelinated had similar g-ratios (reflecting the ratio of the myelin sheath thickness relative to the axon diameter) (93). This suggests that in TH-depleted conditions, some axons might fail to reach a threshold diameter required to induce myelination (95, 96). Importantly, a short T_4_ pulse from postnatal day 15–21 during the period of postnatal hypothyroidism did not restore the myelin deficiency at postnatal day 150 (93), emphasizing the vulnerability of myelin integrity to endocrine changes during this crucial developmental window.

The key role of THs in myelination is also evident in human cases of maternal hypothyroxinemia and congenital hypothyroidism. Children born to hypothyroxinemic mothers have significantly smaller corpus callosums (97), demonstrating that maternal THs during the first half of pregnancy, when the fetal thyroid gland is not yet functional, already promote cellular events that later drive myelination. Children with severe congenital hypothyroidism displayed poorer hearing and communication skills that could be directly correlated to disrupted WM microstructural integrity (72). WM abnormalities were present despite early postnatal treatment with levothyroxine, again underlining the importance of the developmental period for normal myelination (72). Lastly, maternal T_4_ levels during early pregnancy correlate with a higher gray to WM ratio in childhood (98), indicating that reaching optimal WM content requires strictly controlled TH levels. While these studies collectively illustrate that TH signaling during the fetal period is essential for later WM status, myelination is a protracted process particularly in primates (99), expanding the period to which WM development might be susceptible to altered TH levels, or responsive to treatment, up to adolescence.

### A Cellular Lack of MCT8 Could Disrupt Oligodendrogenesis

Knowing how TH promotes the generation of myelinating oligodendrocytes allows understanding how a lack of MCT8 could interfere with this process. Several rodent studies showed how MCT8 expression changes in oligodendroglial lineage cells during development. RNA-Seq analysis on the postnatal mouse brain showed enrichment of *MCT8* mRNA transcripts primarily in OPCs, but also in newly differentiated oligodendrocytes. In more mature myelinating oligodendrocytes, *OATP3A1* was the only potential TH transporter expressed (100). Another transcriptome analysis revealed *MCT10* transcripts in mature oligodendrocytes of the postnatal day 21 mouse brain (101). In the developing rat cerebellum, the myelination markers *OLIG1* and *PDGFR*α were expressed strongly at perinatal stages, while *MBP* mRNA expression and protein levels increased at postnatal day 14 when high levels of circulating THs are detected (21). These temporal expression patterns of important myelin markers coincided with moderately decreased *MCT8* mRNA expression and strongly elevated expression levels of *DIO2* vs. *DIO3* during the first two postnatal weeks, all strictly regulating TH availability (21).

Turning to human evidence, *MCT8* was also enriched in immature OPCs and pre-myelinating oligodendrocytes derived from the NKX2.1-GFP human embryonic stem cell (ESC) line, suggesting MCT8 could enable T_3_ uptake during the maturation phase (90). A transcriptome analysis on human brain cell types also demonstrated moderate *MCT8* expression in mature oligodendrocytes (102). Using immunohistochemistry, one paper suggested MCT8 protein expression in oligodendrocytes of healthy human brain tissue (80). Furthermore, inadequate TH uptake due to the lack of a functional MCT8 protein in cultured human ESCs prohibited OPC cell cycle exit and maturation into oligodendrocytes (80). Hence, it is likely that a lack of MCT8 at the OPC level strongly reduces intracellular T_3_ action, blocking the switch from OPC to mature and myelinating oligodendrocytes (81).

Of note, impaired TH uptake might even occur before TH reaches the oligodendroglial cell membrane. Animal and *in vitro* models of MCT8 deficiency (see section myelin deficiency in animal models of MCT8 deficiency: permanent or delayed?) indicate a crucial role for MCT8 in TH uptake at the endothelial cells of the blood-brain barrier (BBB), and probably also the blood-cerebrospinal fluid barrier (103–106). In addition, one cannot exclude that a lack of MCT8 also affects OPC generation during embryonic development. Unfortunately, no immunohistochemical data were provided for the presence of MCT8 protein in OPCs in a set of prenatal *post mortem* human brains, although MCT8 was widely expressed in several other cell types including radial glia progenitors (107). The latter finding was recently also observed in neural progenitors in human brain organoid cultures (106).

The observation that myelination still progresses in most AHDS patients, albeit at a slow rate, implies that intracellular TH levels might however not be completely depleted in the MCT8-deficient brain, in contrast to deeply hypothyroid brains, which probably never reach a normal WM content (72). Since *in vitro* cellular assays demonstrate that most mutations render the MCT8 protein completely inactive (6), OPCs could be supplied with THs *via* alternative routes. One possibility is *via* the T_4_-selective organic anion transporting polypeptide 1C1 (*OATP1C1*), expressed in radial glial cells (107) for THs supplied by the cerebrospinal fluid during early development (108). *OATP1C1* was not found to be expressed in relevant amounts elsewhere in the human pre- and perinatal brain except for the hypothalamic region (105, 107, 109, 110). Therefore, other transporters, such as MCT10 and the large amino acid transporter 1 (*LAT1*) and *LAT2*, for which expression patterns in the developing human brain are still largely unexplored, might play a role in TH uptake at the OPC membrane itself. If such an alternative oligodendroglial TH transporter(s) exists, it should probably be able to take up T_3_, since the majority of T_3_ for neurons and other cell types is generated by DIO2 in astrocytes (111). Mouse brain OPCs express some *DIO2* (100), but mature oligodendrocytes in the postnatal brain do not (112). So far, there is no data on DIO2 expression in human brain oligodendrocyte lineage cells. Sufficient T_3_ uptake could induce cell-cycle exit and differentiation of some OPCs promoting slow but gradual myelination. An emerging aspect herein is that the oligodendrocyte differentiation potential changes as function of age (113), questioning whether OPCs still respond the same to cues in later life.

Together, these data suggest that MCT8 is a key cellular component driving TH-dependent oligodendrocyte differentiation and myelination. Impaired oligodendrogenesis caused by MCT8 deficiency could therefore functionally impair nerve conduction and thus, contributing to at least some psychomotor problems observed in AHDS.

## Myelin Deficiency in Animal Models of MCT8 Deficiency: Permanent or Delayed?

So far, several genetically altered vertebrates mimic at least some of the characteristics of MCT8 deficiency [for a review, see (114, 115)]. The relevance of mammalian and non-mammalian vertebrate models relies on the fact that TH action during neurodevelopment is highly conserved amongst the vertebrate subphylum (116, 117). The involvement of THs in regulation of the oligodendroglial pathway is universal and conserved throughout evolution (80, 90, 96). Even though some differences might occur *in vivo* due to imperfect sequence homology, and slight alterations in protein stability and substrate selection, MCT8 orthologues of zebrafish, chicken, mice, and humans possess similar transport characteristics (114, 118). The myelin deficit associated with AHDS was explored in some animal models of MCT8 deficiency.

### The Myelin Status in Mouse Models of MCT8 Deficiency

The *Mct8* knockout (KO) mouse was the first available model to study the pathophysiological mechanisms underlying AHDS (119). It reproduces the altered TH serum levels found in AHDS patients, making it an excellent model for the peripheral component of the disease (120). However, *Mct8* KO mice have a normal brain morphology, a normal myelin content as evaluated on frontal brain sections (121), and only mildly altered neurological and locomotor behavior (119, 122). The current consensus is that other TH transporters, notably the highly enriched OATP1C1 at the murine BBB (105), are able to ensure T_4_ uptake that *via* astrocyte-dependent DIO2 provides sufficient T_3_ to neural cells (119, 120, 123). In contrast, OATP1C1 is only very faintly expressed at the human BBB (105). The generation of *Mct8/Oatp1c1* double KO mice was thus essential to replicate the neurological component of AHDS.

Genetic deletion of both *Mct8* and *Oatp1c1* blocked TH uptake by 90%, rendering mice strongly hypothyroid in the CNS, allowing studying the impact on brain structure and function (121). These *Mct8/Oatp1c1* double KO mice displayed pronounced hypomyelination, notably in the corpus callosum as corroborated by the observations of thinner WM tracts (121), consistent with findings in AHDS patients (59). A reduction in FluoroMyelin-stained axons and a lower MBP fluorescent signal intensity on frontal brain sections reflected a hypomyelinated status, suggesting impaired oligodendrocyte differentiation and maturation.

Electron-microscope observations revealed many unmyelinated axons, hampering their conductance, thereby contributing to the neurological deficits and locomotor behavioral impairments (121). The study provided evidence that the myelination deficit is probably permanent given the absence of a normal WM structure at postnatal day 180. Remarkably, the few axons that were myelinated had a normal myelin thickness (121), but whether or not this was directly related to axonal diameter was not investigated. This was also observed in postnatal hypothyroid mice (93), and again indicates that the axonal diameter surpassing a certain threshold is a crucial prerequisite for myelination. This observation suggests that MCT8 deficiency increases the ratio of unmyelinated vs. myelinated axons, but might not affect the myelin sheath itself, i.e., how much myelin per axon is produced. Since ~10% of the normal TH levels were preserved in the brain (121), some oligodendrocytes could thus still take up TH in absence of both transporters, and myelinate axons appropriately. This implies that OPCs express other secondary TH transporters although RNA-Seq analysis of rodent brain cells only showed low expression of several uncharacterized TH transporters (100). Above all, these findings question whether the myelin deficit is a specific problem of a too small axon diameter, or whether is it caused by blocked OPC differentiation, and requires further investigation. Another possibility is that the fraction of oligodendrocytes still able to myelinate under MCT8-deficient conditions represents a unique subpopulation with a specific (epi)genetic signature, or one that functions more independently of MCT8. Purifying these subpopulations and performing high-throughput analysis should provide more answers.

The *Dio2/Mct8* double KO mouse represents another interesting model (124, 125). Following T_4_ uptake by OATP1C1 at the BBB, increased DIO2 activity in astrocytes provides sufficient T_3_ for transport to neurons and oligodendrocytes (126). Therefore, eliminating both genes depletes T_3_ levels in various brain regions, in a slightly different manner than the *Mct8/Oatp1c1* double KO mouse. If OPCs express TH transporters other than MCT8 and OATP1C1, loss of DIO2 might prevent astrocyte-dependent T_3_ supply to cells, resulting in more profound intracellular hypothyroidism. In that case, one can expect a more severe myelin deficiency in *Dio2/Mct8* double KO mice.

### The Myelin Status in the MCT8-deficient Zebrafish

Several zebrafish models were established to investigate the role of TH regulators in development thanks to the zebrafish genetic (127) and physiological similarities with mammalian counterparts, amongst which the thyroid axis [for a review, see (128)]. The myelination process is also strikingly similar to that of mammals. OPCs are generated at day 1 of the 3-day long embryonic development, and following migration form myelinating oligodendrocytes at hatching [i.e., 3 days post-fertilization (3 dpf)]. During this period, a first TH peak occurs (29). Then, myelination starts and myelinated axons are clearly distinguishable in 7 dpf larvae (129, 130). Mct8 is expressed in most oligodendrocytes throughout the zebrafish CNS from 3 dpf onwards (129, 131, 132). The Mct8-deficient zebrafish, first generated by knocking down (131) and then by knocking out *mct8* (129), was the first alternative non-mammalian model for AHDS.

The expression of several genes related to oligodendrogenesis was significantly affected in *mct8*^−/−^ zebrafish. Increased *olig2* expression together with decreased *mbp, p0*, and *plpb1* expression during post-hatch development suggested a block in OPC differentiation to mature oligodendrocytes, corroborated by reduced oligodendrocyte numbers in the CNS, including the spinal cord (129, 132). The oligodendrocytes that still developed in a Mct8-absent environment generated fewer extensions (132), suggesting that associated physiological functions could be impaired. Locomotor activity, sleep-awake cycles and responses to light/dark stimuli were abnormal (129), but could have also been caused by microscopic structural alterations of sensory and motor neurons including reduced axon branching and synaptic density. Thus, it remains unknown to what extent behavioral deficits in these *mct8*^−/−^ fish are a direct consequence of the myelination impairment. An increased number of Schwann cells could indicate that myelination of the peripheral nervous system is hampered too (129). However, the myelin status of nerve axons of the peripheral nervous system has never been explored neither in AHDS patients, nor in any animal model of MCT8 deficiency. Another striking observation was the persistent dysregulated expression of TH-responsive genes related to myelination in the adult *mct8*^−/−^ zebrafish brain (132), though no data is available on oligodendrocyte differentiation and maturation. It is therefore uncertain whether myelination is delayed or persistent in these mutants, although the term hypomyelination is commonly used.

### Future Aspects of Investigating the Myelin Status in Models of MCT8 Deficiency

It is clear that data on myelination phenotypes in animal models of MCT8 deficiency gave preliminary insights into the pathophysiological mechanisms, but so far remain incomplete. We do not know if myelination is delayed or permanent in adult Mct8-deficient zebrafish or 1-year-old *Mct8/Oatp1c1* double KO mice, and the myelin status in other models of MCT8 deficiency still requires further examination. A more thorough investigation of myelination in different CNS regions such as the cerebral cortex and spinal cord are necessary to obtain a more detailed picture of myelin progression. The corpus callosum is often investigated as a reference region for brain WM status, because this large structure is easy discernible and primarily composed of interhemispheric WM tracts. However, it is known that under transient hypothyroid conditions, neural stem cells in the adjacent dorsal subventricular zone generate new OPCs that can populate the corpus callosum and provide functional remyelination in 3-months-old mice (133, 134). It therefore remains uncertain to what extent the myelin status of the corpus callosum reflects the WM status of the entire CNS, especially under MCT8-deficient conditions.

Another major unresolved issue is to what extent the lack of MCT8 in both OPCs and oligodendrocytes and the resulting myelin deficit explain motor and behavioral defects. Lack of TH uptake in global knockouts such as the *Mct8/Oatp1c1* double KO mouse affects nearly all CNS cell types controlling sensorimotor functions, and therefore do not allow addressing how deficient myelination contributes to altered behavior. Use of single cell type genetic technologies such as conditional KO mice or zebrafish lacking MCT8 or both MCT8 and OATP1C1 (135) in OPCs/oligodendrocytes could enable isolating neuronal or neuro-muscular defects caused by pathologies linked to other cell types. Measuring electric potentials across major axonal tracts might also help to identify impaired neuromuscular communication. Models, such as *in vitro* induced pluripotent stem cell-based OPC cultures (136, 137) comparing healthy and MCT8-deficient patients (103), or human ESCs (90) have also been used. To these approaches, one could add Crispr-Cas9 techniques to induce specific gene mutations thereby delivering important insights on oligodendrogenesis and myelination, as well as to identify genotype-phenotype relationships. Another powerful *in vitro* model includes investigating the myelination process in co-cultures of neurons and oligodendrocytes (138).

## Therapeutic Strategies to Ameliorate the Myelin Deficit in MCT8 Deficiency

The neurological symptoms associated with MCT8 deficiency severely affect quality of life and decrease life expectancy. Enhancing myelination is a promising endpoint to alleviate many of the neurological symptoms (80, 90). The therapeutic aim is to restore intracellular TH action in TH-deprived cells, while avoiding side effects in other euthyroid or non-target cells. For OPCs and pre-myelinating oligodendrocytes, this strategy aims to normalize expression of key TH-target genes such as *KLF9, MPB, PLP*, and other candidates to promote proliferation, differentiation and maturation of oligodendroglial cells. Using animal models, different TH analogs could potentially improve or even reverse certain symptoms (104, 129, 139–142). Several of them have also gathered interest as potential therapeutic agents to alleviate the symptoms of AHDS patients. These compounds must meet several *a priori* requirements. They should be taken up independently of MCT8, have thyromimetic actions by binding to nuclear TH receptors α and β, and be metabolized and excreted in a similar fashion to THs (143). However, the transporter(s) responsible for TH analogue in- and efflux remain(s) to be fully identified.

Table 3 summarizes all available data on myelination following the use of TH analogs and other therapeutic strategies in models of MCT8 deficiency and human patients. Some general trends arise from these pilot experiments. First, physiological doses of T_3_ alone were not able to mediate any measurable effects on myelination in *in vivo* models, but remain necessary to confirm the lack of uptake by MCT8 or another secondary transporter. An exception was a high dose of T_3_ that could rescue oligodendrocyte numbers during zebrafish embryonic development (132), probably because a functional BBB was not yet developed at this stage (145). Irrespectively, T_3_ will never provide a therapeutic option due to its severe side effects in off target organs, such as the heart and liver. Second, the dosage of TH analogue(s) is critical. Administration of a high (400 nM) but not a low dose (50 nM) of 3,5,3′-triiodothyroacetic acid (Triac) from postnatal day 1 to 12 to *Mct8/Oatp1c1* double KO mice improved myelination in the cerebral cortex, as analyzed at postnatal day 12 (139). The dose eventually applied in human trials will be a delicate trade-off between beneficial and adverse effects, and also depends on the half-life of a given compound. In the case of Triac, the half-life is ~6 h in humans (142, 146), which limits its therapeutic use, even though it was already successfully used on a long-term in patients with TH receptor β mutations that suffer from the resistance to TH syndrome (147, 148). Three,5,3′,5′-tetraiodothyroacetic acid, or Tetrac, has been proposed as an alternative TH analogue with a half-life in the order of several days (149), but there is far less experience with this drug in animal research and clinical settings. Thirdly, zebrafish experiments showed that the developmental period during which a drug is administered could differentiate between improved oligodendrogenesis or no effect, as already shown above for T_3_. For instance, an *Mct8*-expressing construct in the endothelial cells of the BBB successfully restored oligodendrocyte numbers, but only when it was transfected in later post-hatch development (129, 132).

**Table 3:** Current studies investigating effects of THs, TH analogs and other therapies on the myelination phenotype in models of MCT8 deficiency and human AHDS patients.

**Model**	**Phenotype**	**Drug**	**Dose**	**Window**	**Effect**	**References**
mct8^−/−^ zebrafish	Reduced *p0, olig2, mbp, plp1b* expression Reduced OL numbers Reduced OL extensions Hypomyelination	T_3_	0.5 nM	0–3 dpf	None	(129)
		T_4_				
		Triac			*p0* expression rescued	
		Tetrac				
		Ditpa	5 nM			
		T_3_			Rescue OL numbers	(132)
		Triac				
		Ditpa				
		clemastine (Tavist)	500 nM			
		T_3_	5 nM	6–10 dpf	None	
		Triac			Rescue OL numbers	
		Ditpa			Partial rescue OL numbers	
		clemastine (Tavist)	500 nM			
		*pT2-fli:Mct8-tagRFP* construct to express Mct8 in ECs of the BBB	50 ng/μl	0–10 dpf	No effect at 3 dpf Number of OLs rescued at 10 dpf	
Mct8/Oatp1c1^−/−^ mouse	Severe persistent hypomyelination Many axons unmyelinated Decreased MBP immunoreactivity Reduced WM thickness	Triac	50 ng/g BW/day 400 ng/g BW/day	P1–P12	None Normalization of myelination	(139)
Normal NKX2.1-GFP human embryonic stem cells	None	T_3_	40 ng/mL	21 days	Promotion of cell cycle exit OL differentiation TFs myelin gene expression	(90)
		Ditpa T_3_ + Ditpa	10 ng/mL 10 ng/mL + 40 ng/mL, resp.		Strong promotion of cell cycle exit OL differentiation TFs myelin gene expression	
Normal NKX2.1-GFP human embryonic stem cells in coculture with retinal ganglion cells		T_3_	40 ng/mL	7 days	Far less potent than Ditpa alone	
		Ditpa	10 ng/mL		Increased number of myelinated axons more OL contacting axons amount of myelin/axon unchanged	
		T_3_ + Ditpa	10 ng/mL + 40 ng/mL, resp.		Less potent than Ditpa alone	
MCT8-deprived NKX2.1-GFP human embryonic stem cells in coculture with retinal ganglion cells	OL apoptosis Impaired myelination	T_3_	40 ng/mL	day 3-5 after MCT8-KD	None	
		Ditpa	10 ng/mL		Promotion of OPC survival	
		T_3_ + Ditpa	10 ng/mL + 40 ng/mL, resp.			
		T_3_	40 ng/mL	7 days after MCT8-KD	None	
		Ditpa	10 ng/mL		Increased myelin segments	
		T_3_ + Ditpa	10 ng/mL + 40 ng/mL, resp.			
4 AHDS patients	Delayed myelination at 9 months of age (two twins)	Ditpa	Steady build-up to 3X/day 0.97–1.21 mg/kg BW (Month 4–10) Steady build-up to 3X/day 2.0–2.4 mg/kg BW (Month 15–29)	Started at month 25	Normal myelination 47 months of age	(144)
	Delayed myelination 3, 8, 13 months of age			Started at month 8.5	None*	
	Delayed myelination at 5 months of age			Started at month 25	None*	

*BBB, blood-brain barrier; BW, body weight; dpf, days post-fertilization; EC, endothelial cell; KD, knockdown; MBP, myelin basic protein; OL, oligodendrocytes; Olig2, oligodendrocyte transcription factor; OPC, oligodendrocyte precursor cell; P, postnatal day; p0, Myelin protein zero; plp1b, proteolipid protein 1b; TF, transcription factor; WM, white matter*.

Currently, in animal models there are no evaluations of long-term effects following administration of a TH analogue, nor whether rescuing effects at the cellular level translate into improved functional and behavioral outcomes directly related to improved myelination. Here too, evaluating the effects of TH analogue treatments might be more straightforward in conditional KO models. Another promising approach comprised *in vitro* administration of the TH analogue 3,5-diiodothyropropionic acid or Ditpa, to cultured human NKX2.1-GFP ESCs reprogrammed to become oligodendrocytes (Table 3). Ditpa rescued T_3_-dependent OPC cell-cycle exit and differentiation into oligodendrocytes that were both absent in MCT8-deprived cultures (90). This indicates that T_3_ uptake in human OPCs is strongly MCT8-dependent, and that Ditpa is taken up *via* another TH transporter. Furthermore, Ditpa also inhibited apoptosis of oligodendrocytes and stimulated myelination (90). Importantly, Ditpa crosses the placental barrier in mice (150) showing its potential for prenatal treatment.

The beneficial effects on oligodendrocyte differentiation and myelination of some other treatments that have already been evaluated in other demyelinating diseases [for excellent reviews, see (80, 151)] might hold potential for MCT8 deficiency too, despite different aetiologies. In *Mct8* KO zebrafish, early administration of the antihistamine drug clemastine also partially rescued oligodendrocyte numbers and myelin-related gene expression (132) (Table 3). Similarly, clemastine stimulated OPC proliferation, differentiation and oligodendrocyte maturation in the prefrontal cortex of socially isolated mice that suffer from demyelination, and it also successfully promoted remyelination in animal models of the demyelinating disease MS (152) that is characterized by a loss of myelin-forming oligodendrocytes and locomotor deficits (153).

Another promising drug is sobetirome, also called GC-1, and its CNS-selective prodrug Sob-AM2, a methyl amide derivative (154). The latter crosses the BBB and is converted into the active compound by the CNS-specific enzyme fatty acid amide hydrolase (154), limiting side-effects in peripheral organs. An additional advantage of the prodrug is that it produces increased brain sobetirome levels as compared to equimolar doses of sobetirome (155). Sobetirome is a TH receptor β agonist, previously tested as a drug to combat hypercholesterolemia (156). Sobetirome had myelin-promoting effects by stimulating myelin-associated gene expression and oligodendrocyte differentiation in mouse and human *in vitro* cultures, but also in several mouse brain regions during development (157). The drug was successful in partially remyelinating brain areas in mouse models of MS (158).

Thus, clemastine and sobetirome are two interesting candidates for testing their effects on myelination in MCT8 deficiency models. Sob-AM2 showed already promising thyromimetic effects on gene expression in the brain of *Mct8* KO and *Mct8/Dio2* double KO mice (159). Other drugs already commercially available or in preparation for testing in MS models (160), or stem cell-based treatments proposed for PMD patients (161) might extend to MCT8 deficiency models too. A last promising therapy would only apply to patients carrying *SLC16A2* mutations (e.g., ΔPhe501) that lead to specific MCT8 protein folding defects and reduced uptake activity. *In vitro* experiments showed that the drug phenylbutyrate can stabilize the misfolded MCT8 protein and restore TH uptake (71). It will be interesting to see whether this compound can rescue the myelin deficit and neurological manifestations in mouse models carrying these specific mutations.

Turning to clinical trials, TH analogs have shown potential in AHDS patients to combat the peripheral effects of thyrotoxicosis [e.g., (144, 162) and Triac trial I: NCT02060474], but have so far proven largely inadequate in improving patients' mental status. However, the youngest patients might still benefit from treatment: a clinical trial using Triac in the youngest new-borns is currently underway (Triac trial II: NCT02396459). Other TH analogs were tested in small cohorts of AHDS patients, but only one study included an examination of the myelin status (144). Two out of 4 patients that received increasing doses of Ditpa from 2 years on, showed improved myelination at 47 months of age (144) (Table 3). However, given that myelination improves in many patients during the early years of childhood (Figures 1, 2), the authors could not conclude whether Ditpa was responsible for this progress. Nevertheless, restoring TH action could relieve the quasi-blocked oligodendroglial maturation and myelination at the cellular level, and thus it seems interesting to incorporate this parameter into a more extensive study like the Triac trial that includes more detailed MRI examinations in a larger cohort.

It is also unknown to what extent the embryonic neural stem cell and OPC populations are affected by the central hypothyroidism caused by the mutated MCT8 protein. In mice, the pre- and perinatal period includes the generation of the OPC pool that will persist throughout life within the parenchyma and occurs before and during a postnatal TH peak (29). In the human brain, many cell types express regulators of cellular TH availability from embryonic stages onwards (107). Thus, shifting the treatment window toward a period before birth could increase the efficacy of TH-replacement therapies. Triac has been shown to cross the placental barrier in pregnant women (13), but there are scarce data regarding safety and dose range. In one case, prenatal Triac therapy successfully treated fetal hypothyroidism due to resistance to TH syndrome, although some complications occurred at birth (163). Apart from the risk of major side effects, ethical and methodological issues are raised for human use. Treatments should target and therefore ameliorate the CNS phenotype to avoid worsening the condition of other body organs that suffer from thyrotoxicosis. Furthermore, epidemiological data indicate that subtle hypo- and hyperthyroidism during CNS development can lead to adverse effects on gray matter and WM content (98). Further research along the entire developmental period is necessary to understand the interplay between THs and MCT8 in the oligodendroglial lineage and how that mediates myelination.

## Conclusions

This review underlines the degree of inconsistency existing in the literature on how to classify AHDS amongst the diverse group of WM disorders. One the one hand, this is a consequence of variation amongst patient cases, most showing a gradual improvement of myelination, some still reporting WM abnormalities after several years. On the other hand, MRI studies are not always performed in detail, and follow-up data is often missing. The increasing numbers of case reports in which high resolution, sequential MRIs are performed will progressively give a clearer view on myelin deficits in the disease and whether subtle WM micro-abnormalities are still present later in life. The growing case numbers of AHDS patients identified will also provide more information on patient heterogeneity. Longitudinal assessment of the myelination process in animal models of MCT8 deficiency can further clarify the underlying cellular and molecular mechanisms. Models are also needed to evaluate the translational potential of therapeutic drugs, such as the TH analogs. Here, reciprocal exchanges of new therapeutic evolvements used in other demyelinating diseases sharing some of the hallmarks of AHDS, such as MS or PMD will be particularly edifying. Lastly, comparing data from basic and clinical research assist this same goal, thereby paving the way to improve the health and life span of AHDS patients. Such input will also help those involved in their daily care.

## Author Contributions

PV worked out the concept of the paper and made the figures and tables. PV, BD, and SR wrote the paper.

## Conflict of Interest

The authors declare that the research was conducted in the absence of any commercial or financial relationships that could be construed as a potential conflict of interest.

## Abbreviations

AHDS: Allan-Herndon-Dudley Syndrome
BBB: blood-brain barrier
CNS: central nervous system
DIO: deiodinase
Ditpa: 3,5-diiodothyropropionic acid
dpf: days post-fertilization
ESC: embryonic stem cell
gLE: genetic leukoencephalopathy
KLF9: Krüppel-like factor 9
KO: knockout
LAT: large amino acid transporter
MBP: myelin basic protein
MCT: monocarboxylate transporter
MRI: magnetic resonance imaging
MS: multiple sclerosis
OATP1C1: organic anion transporting polypeptide 1C1
OPC: oligodendrocyte precursor cell
PET: positron emission tomography
PLP: proteolipid protein
PMD: Pelizaeus-Merzbacher Disease
SLC16A2: solute carrier 16A2
T_3_: 3,5,3′-triiodothyronine
T_4_: 3,5,3′,5′-tetraiodothyronine
TH: thyroid hormone
Triac: 3,5,3′-triiodothyroacetic acid
Tetrac: 3,5,3′,5′-tetraiodothyroacetic acid
WM: white matter.
